# Novel Green In Situ Synthesis of ZnO Nanoparticles on Cotton Using Pomegranate Peel Extract

**DOI:** 10.3390/ma14164472

**Published:** 2021-08-10

**Authors:** Anja Verbič, Martin Šala, Ivan Jerman, Marija Gorjanc

**Affiliations:** 1 Faculty of Natural Sciences and Engineering, University of Ljubljana, Aškerčeva 12, 1000 Ljubljana, Slovenia; anja.verbic@ntf.uni-lj.si; 2 National Institute of Chemistry, Hajdrihova 19, 1000 Ljubljana, Slovenia; martin.sala@ki.si (M.Š.); ivan.jerman@ki.si (I.J.)

**Keywords:** food waste, pomegranate, zinc oxide nanoparticles, in situ synthesis, phytosynthesis, cotton

## Abstract

This work presents the novel and entirely green in situ synthesis of zinc oxide nanoparticles (ZnO-NP) on cotton fabric. Pomegranate peel extract was used as a reducing agent and wood ash extract was used as an alkali source for the formation of ZnO-NP from zinc acetate. Four different synthesis methods, which varied in drying between immersion of fabric in the active solutions for synthesis and the use of padding and ultrasonication, were investigated to evaluate the most suitable one to achieve excellent ultraviolet (UV) protective properties of the functionalized textile. For comparison, the cotton fabrics were also functionalized with each active solution separately or in a combination of two (i.e., Zn-acetate and plant extract). Scanning electron microscopy (SEM), inductively coupled plasma mass spectroscopy (ICP-MS), Fourier transform infrared spectroscopy (FTIR), X-ray diffractometry (XRD) analysis, and atomic force microscopy (AFM) confirm the successful formation of ZnO-NP on cotton. Among the synthesis methods, the method that included continuous drying of the samples between immersion in the active solutions for synthesis (Method 4) was found to be the most suitable to deliver uniformly impregnated cotton fibers with numerous small ZnO wurtzite structured crystals and excellent UV protection, with a UV protection factor of 154.0. This research presents an example of a green circular economy where a bio-waste material can be used to produce ZnO-NP directly on cotton at low temperatures and short treatment times without the addition of chemicals and enables the production of cellulosic fabrics with excellent UV protection.

## 1. Introduction

It is widely acknowledged that zinc oxide (ZnO), with its extraordinary photocatalytic activity, chemical stability under UV radiation exposure, thermal stability, and absorption of a broad range UV radiation [1,2] can have broad application in many industries. In the past decade, ZnO has been widely researched also in the textile industry, because when ZnO-NP are applied on textiles, they provide excellent UV blocking, antimicrobial, photocatalytic self-cleaning, hydrophobic, and flame-retardant properties [3,4,5]. There are two main methods of chemical production of ZnO-NP and their application on textiles, i.e., ex situ and in situ synthesis. The ex situ is a two-step process, where in the first step, a synthesis of ZnO-NP is performed as a separate reaction between Zn-precursor and precipitating/reducing agent, followed by calcination, and in the second step, the dispersion of formed ZnO-NP is prepared in a separate bath and coated on textiles [4]. In situ synthesis is a one-step process, in which the synthesis of nanoparticles and their deposition on the surface of the substrate are carried out simultaneously [6,7,8], which leads to a reduction of processing steps and saves energy, time, and cost. Moreover, with ex situ synthesis, very high calcination temperatures are often used, e.g., from 400 to 600 °C [9,10,11], which is not only energy consuming but also not suitable for in situ synthesis on textile substrates, which are sensitive to such high temperatures.

Several hypotheses concerning the mechanism of ZnO synthesis exist. In general, ZnO is expected to crystallize by the hydrolysis of zinc salts in an alkaline solution [12]; therefore, the alkaline conditions play a vital role in ZnO-NP synthesis [6,13]. While the pH value of the synthesis media significantly affects the properties, size, and morphology of the synthesized ZnO-NP [13,14,15,16,17], low pH values are also not suitable for in situ synthesis on cellulosic fabric as it is sensitive to the acidic medium. Moreover, the synthesis of ZnO in an acidic medium (pH ˂ 7) hinders the growth and formation of ZnO due to the low amount of hydroxyl ions (OH^−^) that contribute to the formation of the intermediate molecule zinc hydroxide (Zn(OH)_2_), which facilitates the formation of ZnO [18,19,20]. The majority of green syntheses of ZnO are still performed in the presence of NaOH or KOH, since alkaline pH is required for successful reduction from zinc salt [21,22,23,24]. Additionally, cellulose exposed to an alkaline medium creates more adsorption sites for zinc ions [6,21,25]. When the fabric is subjected to drying and curing processes (increase in temperature), ZnO particles are formed via the dehydration of intermediates [7,12,22,26,27,28]. In addition to reducing agents, different chemical compounds that act as stabilizing agents, dispersing agents, and binders are commonly used in the functionalization process of textile with ZnO as well [21,29]. Since these chemicals can be harmful to human health and the environment [30], researchers are trying to replace them with more environmentally friendly compounds, such as microbes or phytochemicals [31]. Phytosynthesis is a method in which phytochemicals present in plant extracts are used as reducing, capping, and stabilizing agents in nanoparticle formation [31,32,33,34,35,36]. The process is still not fully understood, but it is believed that primary and secondary metabolites are responsible for the synthesis of metal nanoparticles and act as reducing and stabilizing agents. While phenolic compounds have high antioxidant potential and are very good reducers of metal ions [28,36,37], carboxyl groups are supposed to stabilize ZnO-NP [38]. Some of the researchers observed that the type of natural extract used in the synthesis process could affect the morphological, optical, and photocatalytic properties of ZnO-NP [35], although these nanoparticles were not synthesized directly on textile substrates. It is suggested that a higher concentration of natural extracts contains a higher number of capping agents, resulting in more stable nanoparticles that do not agglomerate [31].

There are a few published studies on the green ex situ synthesis of ZnO and their application on textile substrates where bacterial extracts [26,39], Pongamia pinnata leaves [27], Rambutan peels [28], Yoshino cherry leaves [36], and Acalypha indica extract [9] were used. However, many studies in the preparation of ZnO-NP still include either classical reducing agents (i.e., NaOH, KOH) or the preparation of extracts in organic solvents and therefore cannot be stated as truly an environmentally benign green synthesis [21,22]. There are studies published in which the syntheses of ZnO were performed with only zinc precursor and natural plant extract, but the nanoparticles were calcinated at very high temperatures (up to 600 °C) [9,40], which makes this procedure unsuitable for in situ synthesis on cotton fabric, as the cellulose is sensitive to such high temperatures, and it would degrade.

In the literature review of green in situ synthesis of ZnO-NP on textiles, we found many papers where the title included these keywords, but from the content of the papers, it was clear that the process was either not in situ or it was not truly green, as classical chemicals were still used in the synthesis process. From this, it is clear that methods for truly green in situ synthesis on textiles is not an easy task to be achieved. To our knowledge, there is only one research where ZnO-NP were synthesized in situ on cotton fabric using a natural extract and without the addition of other chemicals [41]. In the study, Keliab, the ashes of burnt leaves and stems of Seidlitzia Rosmarinus plant, was used as an alkali source, containing large amounts of sodium and potassium carbonates. ZnO-NP were in situ synthesized on raw cotton fabric, using zinc acetate dihydrate solution as a zinc precursor. The fabric was immersed in the precursor solution for 30 min under continuous stirring. Then, the Keliab solution was slowly dripped into the synthesis bath, and the temperature was elevated to 90 °C. The synthesis process was continued for the next 60 min. Then, the samples were dried at 80 °C for 30 min and cured at 150 °C for 3 min. The functionalized samples showed good antimicrobial properties against *Staphylococcus aureus* and *Escherichia coli*, enhanced tensile strength, and higher crease recovery compared to untreated samples. Although good results were obtained, the synthesis process was still time and energy consuming, as it was performed for 90 min and at high temperatures. Moreover, the plant used for the preparation of natural extract is not a waste material and can be used for other purposes. This type of process is not an example of green circular economy, since if it were produced on an industrial scale, the demand for growing this plant would increase dramatically. Summarized key parameters on green synthesis of ZnO are presented in Table 1.

The sustainable and cost-effective source of phytochemicals is under-utilized inedible by-products of food production, i.e., peels, seed, and leaves of various plants, which represent a major environmental burden [42]. The pomegranate market has very high consumer demand worldwide, as the global production is estimated to 2,000,000 metric tons annually [43]. According to research reports [44], the market is expected to gain growth in the following years. Due to the high consumption of this edible plant, a high amount of inedible food waste (peel) is generated, which makes it an excellent candidate for utilization in the textile industry, where it can be used as an environmentally friendly source of phytochemicals for the functional finishing of textiles. The phytochemicals extracted from the food waste are also excellent candidates to solve the problem of using harsh chemicals in the ZnO synthesis and textile application process.

In this paper, we present an innovative and green approach for the in situ (direct) synthesis of ZnO-NP on cotton fabric, which would provide excellent protection against harmful UV radiation, by using a zinc precursor and only natural water extracts from waste, without other chemicals. Water extract from pomegranate peel was used as the source of reducing agent, and wood ash extract from burnt wood pellets was used as the source of alkali. In addition, different synthesis methods were investigated to gain insight into the most suitable method. The formation of ZnO on cotton fabric and its properties were evaluated by SEM, FTIR, ICP-MS, AFM, XRD, measurement of UV protection factor, and antioxidant activity.

## 2. Materials and Methods

### 2.1. Materials

Plain weaved, chemically bleached, and mercerized cotton (weight: 110 g/m^2^, fabric thickness: 0.201 mm, number of warp threads: 60 threads/cm, number of weft threads: 32 threads/cm), (Tekstina d.d., Ajdovščina, Slovenia) and zinc acetate dihydrate (C_4_H_6_O_4_Zn·2H_2_O, Honeywell, Charlotte, NC, USA) were used in this study.

### 2.2. Preparation of Natural Extracts

**Figure 1:**
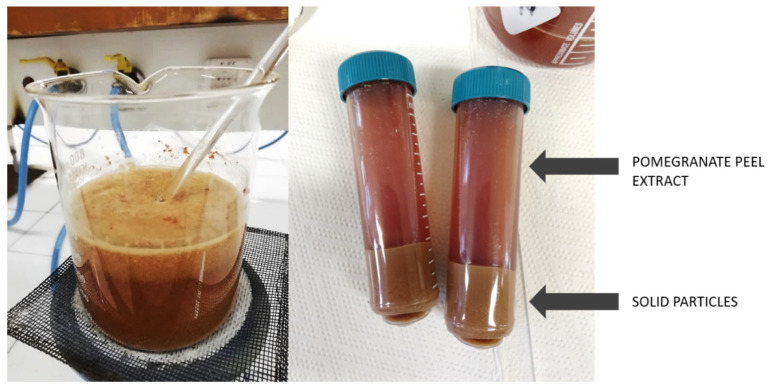
Preparation of pomegranate peel extract: heating the solution (**left**) and separation of solid particles from the extract with centrifugation (**right**).

Pomegranate fruit was collected from the local supermarket (Zeleni Hit d.o.o., Ljubljana, Slovenia), where the fruit was discarded due to not meeting the retail standards. The peels were separated from the seeds of the pomegranate fruits, and then the peels were washed, cut into small pieces, and air dried. The dried peels were ground into powder using a kitchen blender (Tefal, Rumilly, France). The extracts were prepared by immersing 100 g/L of powder into bidestilled water. The temperature of the solution was increased to boiling point, held at that temperature for 5 min and left to cool for two hours. Then, the solution was centrifuged at 4000 rpm to separate the solid particles from the liquid (Figure 1), which was further filtered through PES mesh to remove the remaining particles and thus obtained pure liquid extracts. The extracts thus prepared were used immediately.

**Figure 2:**
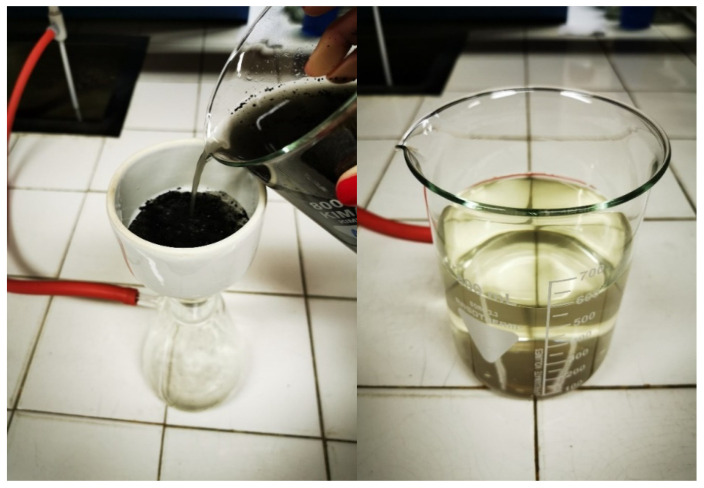
Preparation of wood ash extract: vacuum filtering the solution (**left**) and wood ash extract (**right**).

To ensure alkaline medium for the in situ synthesis of ZnO, we used a natural extract prepared from wood ash waste. The ash of burnt commercial wood pellets, used for the heating system, was collected and used for the preparation of water extract without any additional modification or purification. Wood ash extract was prepared by immersing 10 g/L of ash powder into bidistilled water. The mixture was vacuum filtered after 5 min (Figure 2). The pH of the prepared wood ash extract was measured immediately after preparation, and its value was 10.44.

### 2.3. In Situ Synthesis of ZnO on Cotton Fabric

**Figure 3:**
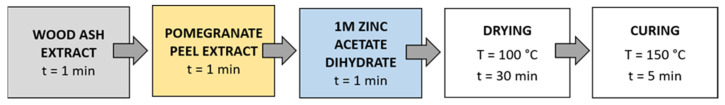
Schematic presentation of Method 1 of in situ synthesis of ZnO-NP.

The ZnO-NP were in situ synthesized on cotton fabric using four different methods, as described in detail below:

Method 1 (Figure 3): The samples were first immersed in a wood ash extract for 1 min, then in a pomegranate peel extract for 1 min, and in a 1 M zinc acetate dihydrate solution for 1 min. Then, the samples were dried in a continuous dryer for 5 min, which was followed by drying in a laboratory oven at 100 °C for 30 min and curing at 150 °C for 5 min.

**Figure 4:**
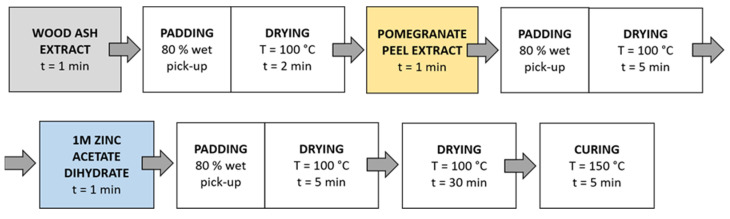
Schematic presentation of Method 2 (pad-dry) procedure of in situ synthesis of ZnO-NP.

Method 2 (Figure 4): Samples were immersed in wood ash extract for 1 min, passed through a two-roll laboratory padder (Werner Mathis, Zurich, Switzerland) with a wet-pick-up of 80%, and dried in a continuous dryer for 2 min; then, they were immersed in pomegranate peel extract for 1 min, passed through a laboratory padder, dried in a continuous dryer for 5 min, and finally immersed in a 1 M zinc acetate dihydrate solution for 1 min, passed through a laboratory padder, and dried in a continuous dryer for 5 min. Lastly, the samples were dried in a laboratory oven at 100 °C for 30 min and cured at 150 °C for 5 min.

**Figure 5:**
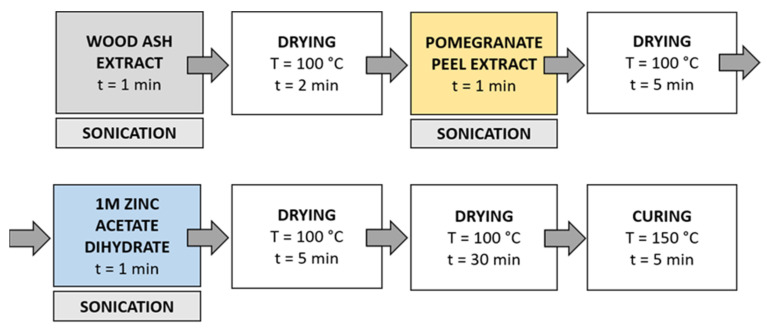
Schematic presentation of Method 3 (ultrasound) procedure of in situ synthesis of ZnO-NP.

Method 3 (Figure 5): in this method, the synthesis solutions were sonicated while the samples were immersed in the solution. Samples were immersed in wood ash extract for 1 min during sonication and dried in a continuous dryer (KTF-500, Mathis, Zurich, Switzerland) for 2 min; then, they were immersed in pomegranate peel extract for 1 min during sonication and dried in a continuous dryer for 5 min, and finally, they were immersed in a 1 M zinc acetate dihydrate solution for 1 min during sonication and dried in a continuous dryer for 5 min. Lastly, the samples were dried in a laboratory oven at 100 °C for 30 min and cured at 150 °C for 5 min.

**Figure 6:**
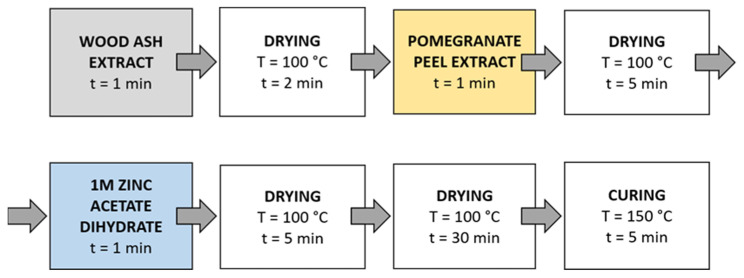
Schematic presentation of Method 4 of in situ synthesis of ZnO-NP.

Method 4 (Figure 6): The samples were first immersed in a wood ash extract for 1 min, which was followed by drying in a continuous dryer for 2 min. Then, the samples were immersed in a pomegranate peel extract for 1 min and dried in a continuous dryer for 5 min. Next, the samples were immersed in a 1 M zinc acetate dihydrate solution for 1 min and dried in a continuous dryer for 5 min. Then, all samples were dried in a laboratory oven at 100 °C for 30 min and at 150 °C for 5 min.

### 2.4. UV Protection Factor Measurements

The ultraviolet protection factor (UPF) of the untreated and functionalized cotton fabric samples was determined according to the AATCC TM 183 standard. Measurements were performed using a Varian CARY 1E UV/VIS spectrophotometer (Varian, Melbourne, Australia) containing a DRA-CA-301 integration sphere and Varian Cary WinUV software (Varian, Melbourne, Australia). The transmissions of ultraviolet radiation through samples were measured in the spectral range between 280 and 400 nm, and the average transmittance (T) at wavelengths between 315 and 400 nm (UVA), 280 and 315 nm (UVB), and 280 and 400 nm (UVR) were determined. The UPF was calculated by Varian Cary WinUV software (Varian, Melbourne, Australia) according to the following equation:

(1) $$\mathrm{UPF}=\frac{\sum_{\lambda =280}^{400}\cdot E\lambda \cdot S\lambda \cdot \Delta \lambda }{\sum_{\lambda =280}^{400}\cdot E\lambda \cdot S\lambda \cdot T\lambda \cdot \Delta \lambda }$$

where Eλ is the relative erythermal spectral effectiveness, Sλ is the solar spectral irradiance, Tλ is the spectral transmittance of the specimen, and Δλ is the measured wavelength interval in nm. The UPF rating was automatically calculated by Varian Cary WinUV software (Varian, Melbourne, Australia) averaging 6 measurements of each sample, and the protection category was determined according to the Australian/New Zealand Standard: Sun protective clothing—Evaluation and classification. The Australian/New Zealand Standard (AS/NZ 4399:2017) defines criteria for assessing the UV protective effectiveness of textiles and evaluation for labeling textile products with a protective function. The standard classifies textile products into three categories of protection, namely, excellent, very good, and good protection. The values are in the range of 15 to 50, and the higher the value, the better the protection. Values lower than 15 are considered insufficient UV protection, between 15 and 30 are considered minimum UV protection, between 30 and 50 are considered good UV protection, and above 50 are considered excellent UV protection.

### 2.5. Scanning Electron Microscopy (SEM)

The morphology of the untreated and functionalized cotton samples was observed using JSM-6060 LV (JEOL, Tokyo, Japan) scanning electron microscope. Prior to SEM analysis, the samples were coated with a layer of gold to ensure sufficient electrical conductivity. SEM microscopic images were taken at 5000× magnification.

### 2.6. Fourier Transform Infrared Spectroscopy (FTIR)

ATR-FTIR spectra of untreated and functionalized cotton samples were acquired using a Bruker Optics Vertex 70 FTIR spectrometer purged by N_2_, equipped with a Bruker Platinum ATR single reflection diamond accessory, using a Ge on KBr substrate beamsplitter and a liquid nitrogen-cooled wide band mercury cadmium telluride (MCT) detector. The spectra were recorded over the range 4000–400 cm^−1^, with a resolution of 4 cm^−1^. The average was obtained for 128 spectra.

### 2.7. Inductively Coupled Plasma Mass Spectroscopy (ICP-MS)

ZnO-functionalized cotton samples were analyzed by mass spectrometry with inductive coupled plasma after microwave digestion. All reagents used were of analytical grade or better. For sample dilution and the preparation of standards, ultrapure water (MilliQ, Millipore, Burlington, MA, USA) and ultrapure acids (HNO_3_ and HCl, Merck Suprapur, Darmstadt, Germany) were used. Standards were prepared in-house by the dilution of certified, traceable, inductively coupled plasma (ICP)-grade single-element standards (Merck CertiPUR, Darmstadt, Germany). Prior to ICP-MS analysis, each sample was weighed (approximately 100 mg) and digested using a microwave-assisted digestion system (CEM MDS-2000, CEM, Matthews, NC, USA) in a solution of 7 mL of nitric acid and 2 mL of hydrogen peroxide. The digested samples were cooled to room temperature and then diluted with 2% *v*/*v* nitric acid until their concentration was within the desired concentration range and were used for subsequent analyses. An Agilent Technologies (Agilent Technologies, Santa Clara, CA, USA) 7500ce ICP mass spectrometry (MS) instrument, equipped with a MicroMist glass concentric nebulizer, and Peltier-cooled, Scott type spray chamber was used. Three measurements per each synthesis method were performed, and the average value and relative standard deviation was calculated.

### 2.8. Color Measurements

A reflectance spectrophotometer Spectraflash 600 PLUS-CT (Datacolor, New Jersey, USA) was used for measuring CIE L*a*b* values of the samples, where L* indicates the lightness of the sample, a* is a red/green coordinate, and b* is a yellow/blue coordinate. The color measurements were performed with a 3 mm aperture using four fabric layers. The specular component of the spectrophotometer was included. Five measurements were made on each sample, and the average was calculated automatically by the Datacolor software.

The color strength (K/S values) from the reflectance measurement was calculated based on Equation (2):

(2) $$\frac{K}{S}=\frac{{\left(1-R\right)}^{2}}{2R}$$

where R represents the reflectance, K means the absorbance, and S represents the scattering of the sample.

### 2.9. Antioxidant Activity (DPPH Assay)

The antioxidant activity of cotton samples was analyzed with the 1,1-diphenyl-2-picrylhyrazyl (DPPH) free radical scavenging method [45,46,47]. Cotton samples were allowed to react with 0.1 mM ethanolic solution of DPPH for 30 min at 37 °C in the dark under shaking conditions. The absorbance of the reaction solutions at 517 nm was measured using Varian Cary 1E UV/VIS spectrometer (Varian, Melbourne, Australia) two times for each sample, and the average value was calculated. The standard deviations of the measurements are presented in results table. The antioxidant activity was calculated according to the equation below.

Antioxidant activity (%) = [(Ac − As)/Ac] × 100,

where Ac is the absorbance of blank DPPH solution and As is the absorbance of DPPH solution in contact with functionalized cotton fabric [45].

### 2.10. Determination of Total Phenolic Content (TPC)

The Folin–Ciocalteu method was used to determine the total phenolic content of natural extracts [48]. Clear supernatants (0.5 mL) were transferred to test tubes and Folin–Ciocalteu agent (Sigma Aldrich, St. Louis, MO, USA) (2.5 mL) was added to each tube. After 3 min, 2 mL of 20% Na_2_CO_3_ solution was added, and the mixture was kept in dark for 2 h. Absorbance values were measured by a UV-Vis spectrophotometer (Lambda 25, Perkin Elmer, Waltham, MA, USA) at 760 nm. A linear calibration curve was obtained with gallic acid, and results were expressed as mg gallic acid equivalents (GAE) per 100 g of the sample.

### 2.11. Determination of Total Flavonoids Content (TFC)

According to the method proposed by Zhishen et al. [49], catechin solution (20, 40, 60, 80, and 100 mg/L) was used as standard in the calibration curve. First, 1 mL of the prepared extract or standard was mixed with 4 mL of distilled water and 0.3 mL of 5% NaNO_2_. After 5 min, 0.3 mL of 10% AlCl_3_ was added and after 1 min, 2 mL of 1 M NaOH was added. Then, 2.4 mL of distilled water was immediately added, and the mixture was shaken. The pink color formed was read using a UV/VIS spectrophotometer (Lambda 25, Perkin Elmer, Waltham, MA, USA) at a wavelength of 510 nm, and the results were expressed as mg catechin equivalent (CE) per 100 g of the sample [48].

### 2.12. Atomic Force Microscopy (AFM)

The topography of the studied samples and the influence of the functionalization on the morphology of the fabric was evaluated using an atomic force microscope AFM WITec alpha 300 RAS (Ulm, Germany). Surface scans of 10 μm^2^ square areas were performed by AFM Arrow Cantilever Reflex coated—FM(AC), 2.8 N/m, 75 kHz.

### 2.13. X-ray Diffractometry (XRD)

X-ray diffraction patterns of samples were recorded on an X-ray diffractometer (XRD) PANalytical X’Pert PRO (Malvern, UK) (CuKα1 = 1.5406 Å) with a completely open X’Celerator detector (2.122° 2 θ). The XRD pattern was measured from 4 to 80° 2 θ with a step size of 0.034° 2 θ and 100 s integration time.

## 3. Results

**Figure 7:**
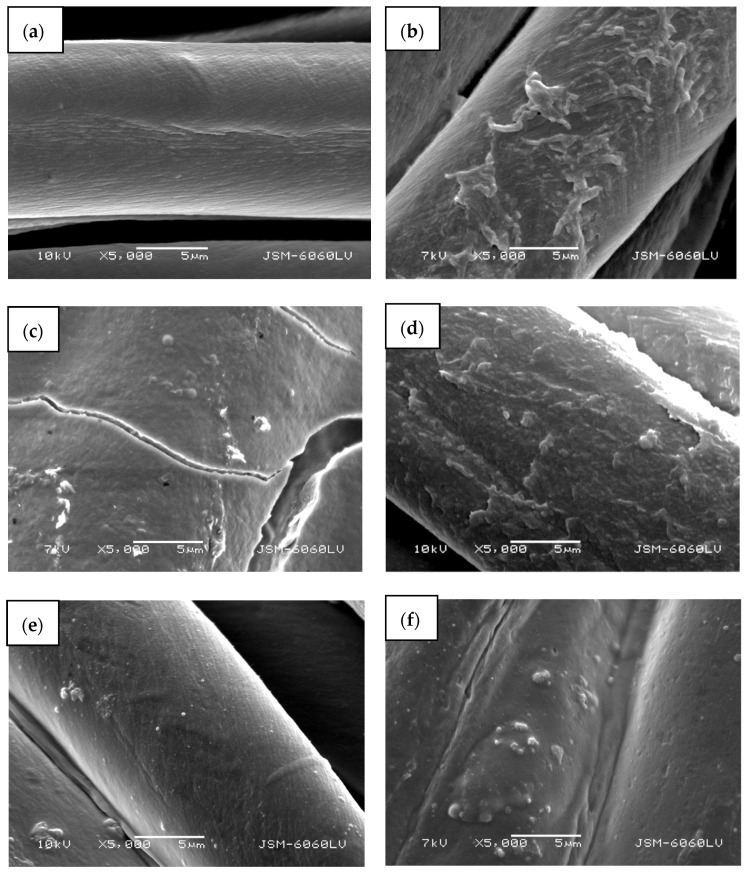
SEM images of cotton samples: (**a**) untreated, (**b**) functionalized with zinc acetate dihydrate, (**c**) functionalized by Method 1 synthesis, (**d**) functionalized by Method 2 synthesis, (**e**) functionalized by Method 3 synthesis, and (**f**) functionalized by Method 4 synthesis at 5000× magnification.

A completely green method of ZnO in situ synthesis on cotton fabric using pomegranate peel extract as a reducing agent and wood ash as alkali source is presented. Different synthesis methods were applied (i.e., with or without drying between impregnation, additional padding, or ultrasonication) to evaluate the most appropriate. To eliminate the influence of temperature on the formation of ZnO nanoparticles using only a precursor, the cotton samples were also functionalized with precursor only and heated. The scanning electron microscopy images (Figure 7) show different morphologies of the cotton fibers. The untreated cotton sample (Figure 7a) has a typical morphology of mercerized cotton, and the fibers’ rather smooth surface is noticeable.

Treating the cotton sample with zinc acetate dihydrate solution leads to the formation of net-like structures on the fibers’ surface (Figure 7b), which can be attributed to the formation of crystalline form of Zn-acetate [50]. Here, no individual ZnO particles or crystals were formed and are not visible on the fibers. The cotton sample functionalized with Method 1 of in situ synthesis of ZnO-NP, where no padding or drying was performed between impregnation with the active solutions for synthesis, results in the formation of a thick layer on the fabric and fibers’ surface with individual ZnO-NPs (Figure 7c). Due to the thickness of the formed layer, cracking is visible on and between the fibres. The sample also had a stiffer hand-feel than the other samples. The synthesis of the ZnO-NP on cotton by Method 2, using the pad-dry procedure between immersion into active solutions for the synthesis, shows an evenly distributed coating on the fibers (Figure 7d), which is quite similar in morphology to the sample treated with the precursor only (Figure 7b). However, some differences can be observed, such as the formation of individual clusters and particles, which can be attributed to formed ZnO-NP. A very smooth surface with many individual, evenly distributed particles can be seen on a sample where Method 3 was used for the functionalization of cotton fabric (Figure 7e). The method involved the ultrasound process and drying between impregnation with active solutions for synthesis. The functionalization of cotton fabric using Method 4, which included drying in a continuous dryer between the immersion in the synthesis solutions, results in the uniformly coated cotton fibers, with visible numerous small ZnO particles and their agglomerates (Figure 7d).

**Figure 8:**
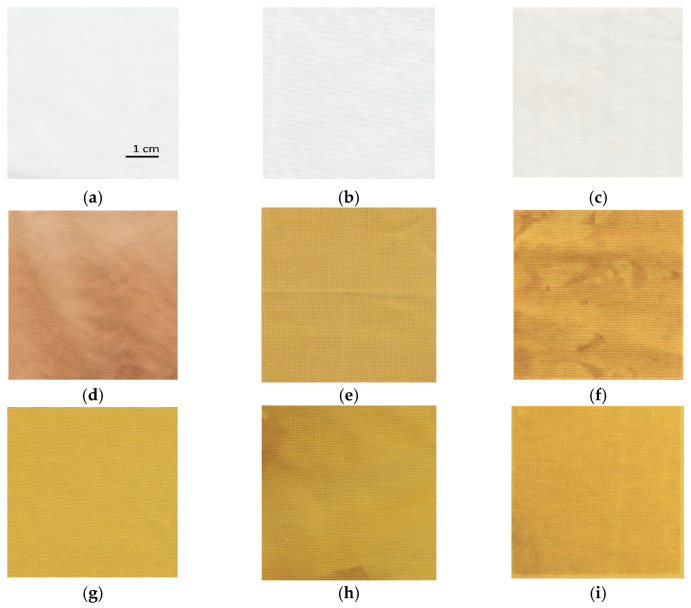
The images of scanned cotton samples: (**a**) untreated, (**b**) functionalized with zinc acetate dihydrate, (**c**) functionalized with wood ash extract, (**d**) functionalized with pomegranate peel extract, (**e**) functionalized with pomegranate peel and zinc acetate dihydrate, (**f**) functionalized by Method 1 synthesis, (**g**) functionalized by Method 2 synthesis, (**h**) functionalized by Method 3 synthesis, (**i**) functionalized by Method 4 synthesis.

The influence of different synthesis methods on color properties was analyzed. The scanned samples are presented in Figure 8. The color values (CIE L*a*b*) are presented in Table 2. The lightness of the untreated cotton sample is relatively high, with a value of 91.66. The sample has a slight green and yellow undertone. Treating the sample with zinc acetate dihydrate solution does not noticeably change the color values. While the untreated cotton fabric and sample treated with only zinc acetate dihydrate solution have slightly green (a* = −0.27 and −0.49) and yellow undertones (b* = 1.01 and 1.80), samples functionalized with pomegranate peel extract become more red and yellow. Treating the cotton with wood ash extract has no significant effect on the lightness of the sample (L* = 88.72) or the value of the green/red axis (a* = 0.14). The value of the blue/yellow coordinate shifts toward yellow (b* = 5.85). Pomegranate peel extract darkens the sample (L* = 70.36). The cotton sample treated with pomegranate peel extract and zinc acetate dihydrate results in almost the same lightness value as treating it with pomegranate peel extract alone, while a shift toward the green is observed (a* = −2.50). All samples where in situ synthesis was performed have significantly lower lightness values (from 56.16 to 71.36), whereas there is also a major shift visible at both green/red and blue/yellow color coordinates due to the presence of pomegranate peel extract. The increase in red undertone is the most significant for the sample where only pomegranate peel extract was used (a* = 7.93), while the value of green/red coordinate decreases for the samples where in situ synthesis was performed (from −1.77 to 5.73). While all samples in which pomegranate peel extract was used exhibit more pronounced red undertone, using Method 2 of in situ synthesis results in a greener undertone of the sample, although this difference is not visible to the naked eye. The blue-yellow coordinates of the samples where pomegranate peel extract was present in the functionalization process increase dramatically. While the sample functionalized only with pomegranate peel extract has a CIE b* value of 34.92, the yellow coordinate even increases for the sample where in situ synthesis was performed (from 47.17 to 55.61). This color transition is visible to the naked eye during the synthesis process. When the sample is immersed to zinc acetate dihydrate solution, the color immediately changes to bright yellow. According to the literature [36,51], the color transition to bright yellow or yellowish green could be a confirmation that ZnO nanoparticles have been synthesized.

The K/S values of the untreated and functionalized samples are presented in Table 3. The color strength of the untreated cotton sample and sample, treated with only zinc acetate dihydrate solution is very low, with a value of 0.04 and 0.05, respectively. Treating the sample with wood ash extract also does not increase the K/S value notably. When pomegranate peel extract is used in the functionalization process, the color value increases significantly. When using the extract alone, the K/S value increases to 10.85. Treating the cotton with pomegranate peel extract and zinc salt results in a lower K/S value than using the extract alone (K/S = 9.09). Using Method 1 of in situ synthesis of ZnO-NP results in the highest K/S value of 12.37. As observed by SEM, this sample has an uneven and thick coating covering the fibers. High K/S values mean that the samples contain the highest amount of the colorant [52]; i.e., the highest amount of natural extract was adsorbed. The sample where the first step of immersing the cotton in wood ash extract was excluded and the sample was treated only in pomegranate peel extract and zinc acetate dihydrate exhibits the lowest K/S value among the samples functionalized with pomegranate peel extract. Not all hydroxyl groups in the cellulose chain molecule are equally accessible to water, chemical reagents, or dyes [53,54]. Alkaline media increases the amount of free OH^–^ functional groups due to the swelling, giving a more open accessible structure [55], which enables the cellulose substrate higher adsorption of plant extract and therefore increases the K/S value of the cotton fabric. The high K/S value is also observed with the sonicated sample (Method 3). When the padding was included in the functionalization process, the lowest K/S value was obtained. According to the CIE L*a*b* measurements, this sample has the highest lightness value of all in situ synthesized samples, which means that the padding process could excessively remove the synthesis solutions from the fibers after immersion. The K/S value obtained by Method 4 is 10.98, which is second lowest among samples functionalized by different synthesis methods.

**Figure 9:**
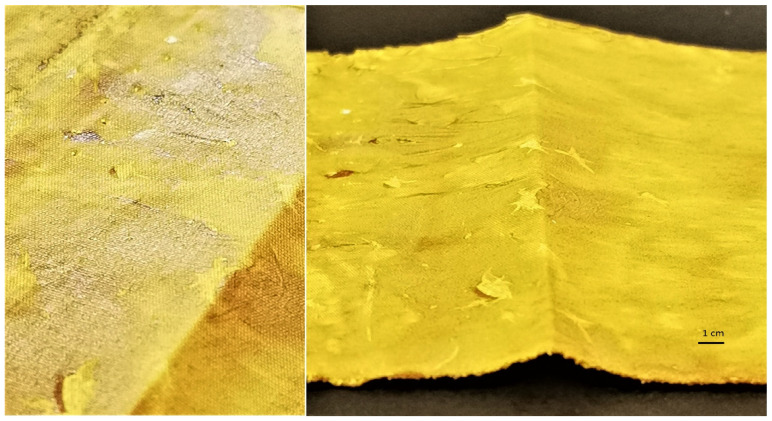
Images of the cotton sample functionalized by Method 1 of in situ synthesis of ZnO.

The UV protective properties of the functionalized samples were analyzed further. The ultraviolet protection factor (UPF), transmission of UVA and UVB radiation (T(UVA) and T(UVB)), UVA and UVB blocking and protection category of the untreated cotton sample, the sample treated with pomegranate peel extract only, and the samples where in situ synthesis of ZnO was performed using Methods 1−4 are presented in Table 4. The untreated, chemically bleached, and mercerized cotton sample has insufficient protection against UV radiation, with a UPF value of 3.9, which means that this sample transmits 27.3% of UVA + UVB radiation and blocks 71.1% (UVA) and 75.2% (UVB) radiation. Treating the cotton sample with wood ash extract does not significantly increase the UPF value (UPF = 6.9); therefore, functionalizing the fabric in alkaline medium alone does not increase the UV protection of the sample, since the fabric still transmits 20.7% of UVA and 16.5% of UVB radiation. Functionalizing the cotton sample with pomegranate peel extract drastically increased the UPF value to 88.6. The values of UV transmissions are much lower than with the untreated or pomegranate peel extract treated cotton, since the sample transmits only 1.5% of UVA and 1.2% of UVB radiation. This shows that pomegranate peel extract alone has an excellent UV blocking ability. Pomegranate extracts contain organic compounds (flavonoids, polyphenols) that act as UV absorbers, but also as strong antioxidants [56,57]. When the cotton sample was treated with pomegranate peel extract and zinc acetate dihydrate solution, the measured UPF value of the cotton sample was 50.7. While this sample achieves excellent UV protection according to the Australian/New Zealand Standard, the UPF value is actually lower than with the sample functionalized with pomegranate peel extract only (treating the fabric with only pomegranate peel extract provides better UV protection). These results again show that the addition of alkaline medium in the synthesis process is crucial for the successful synthesis of ZnO-NP and consequently high UV protection, and that pomegranate peel extract and zinc acetate dihydrate alone are not enough for in situ synthesis of ZnO to take place. The results of the UV/VIS spectrometry measurements of in situ synthesized ZnO-NP on cotton fabric using pomegranate peel with Methods 1−4 shows that regardless of the method used, all samples provide excellent UV protection according to the AS/NZ 4399:2017 standard, as the UPF values range from 92.3 up to 1083.9. All samples where in situ synthesis of ZnO was performed (Methods 1−4) have especially high UV radiation blocking ability, with the values from 98.5% to 99.8% (UVA) and 98.9% to 99.9% (UVB). The color measurement values showed that the samples with very high K/S values (pomegranate peel treated cotton and pomegranate peel and zinc acetate dihydrate treated cotton) do not have the highest UV protection, proving that UV protection of our samples is not color-dependent. The excellent UV protection is attributed to the formation of ZnO-NP on cotton, which is only achieved with in situ synthesis of ZnO-NP. The highest UV protection is achieved with Method 1 (UPF = 1083.9), which is attributed not only to the formation of ZnO-NP, but also to the thick layer covering the fabric (Figure 9). Although this sample provides outstanding UV protection with 99.8% UVA and 99.9% UVB radiation blocked, the method used for in situ synthesis on cotton fabric is not applicable for a practical use; as mentioned previously, the fabric was very stiff and therefore not suitable for handling. For this reason, this sample was excluded from further study.

The inclusion of the pad-dry process in the functionalization process results in a decrease of the UPF value of the cotton fabric. The UPF value is 92.3, which is the lowest of all the in situ synthesis methods used. The reason is a removal of the active compounds for synthesis during padding and therefore their lower ability to form ZnO-NP on cotton. During the experiment, layers of solutions were observed on the padder cylinder. Excluding the padding phase and leaving sonication and drying phases (Method 3) or only the drying phase (Method 4) in between the impregnation of the fabric with active compounds for synthesis results in much better fabric handling and uniform impregnation with ZnO-NP on the fibers’ surface (Figure 8h,i) and consequently higher UPF values (Table 4). However, with Method 4, a much higher UPF value is achieved (154.0), which means that the cotton sample blocks 99.0% of UVA radiation and 99.2% of UVB radiation.

**Figure 10:**
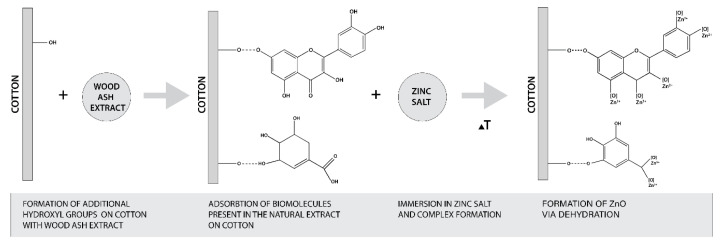
Schematic presentation of proposed mechanism of in situ synthesis of ZnO on cotton.

The formation of ZnO from zinc salt is governed by the hydroxyl groups of plant extract polyphenols [27,35]. Regarding the pomegranate peel extracts, it was found that high values of phenolic and flavonoids compounds in the extract were directly correlated to the reducing power of iron (III) to iron (II) [58]. The content of phenolic and flavonoids compounds depends on the concentration of the extract, time and temperature of extraction, and the solvent used [58,59,60,61]. Therefore, it was essential to evaluate the total phenolic content (TPC) and total flavonoid content (TFC) of our extract. The results are presented in Table 5 and show that the TPC content was 2911.8 mg GAE/100 g and the TFC content was 765.8 mg CE/100 g. Comparing our results with those obtained in organic solvents extraction (i.e., TPC of 510 mg GAE/g, TFC of 16.4 mg quercetin/g in 60% ethanol extract [61], TPC of 124.34 mg GAE/g, and TFC of 59.44 mg quercetin/g in methanol extract [58], it is clear that the preparation of the extract in water enables very high TPC and TFC values and therefore was able to reduce the Zn precursor to ZnO-NP. Considering that the alkaline medium increases the amount of free OH^−^ groups of cellulose, which allows higher adsorption of phytochemicals from plant extract, and consequently increases the amount of phenolic and flavonoid compounds on cotton fabric, this favors the formation of ZnO-NP on cotton. The proposed mechanism of in situ formation of ZnO on cotton was adapted from the published research of Anandan (2019) and Matinise (2017) [62,63] and is schematically presented in Figure 10.

Many studies have reported that plant extracts exhibit high antioxidant activity [64,65,66], and it was also found that there is a direct correlation between antioxidant activity and the reducing power of pomegranate peel extract [58]. Therefore, the polyphenolic compounds of the extract are consumed for the formation of ZnO. To confirm the reducing power of pomegranate peel aqueous extract, the antioxidant activity of the extract and functionalized cotton fabrics were evaluated (Table 6). The untreated and zinc acetate dihydrate-treated cotton fabrics have negligible antioxidant activity, with values of 0.7% and 0.4%, receptively. Pomegranate peel extract exhibits excellent antioxidant activity with a value of 94.8%. Cotton fabric functionalized with only pomegranate peel extract exhibits lower antioxidant activity than extract alone, with a value of 79.7%, which is due to the partial consumption of phytochemicals present in the extract for adsorption on cotton. When in situ synthesis of ZnO-NP is performed on cotton, the samples exhibit significantly lower values of the antioxidant activity, with values ranging from 25.9 to 40.8%. The decrease of antioxidant activity of cotton samples is due to the consumption of polyphenolic compounds for adsorption on cotton but predominantly for the formation of ZnO-NP, which additionally confirms the formation of ZnO on cotton. Mahendra et al. [67] also found that the antioxidant activity was highest for plant extracts alone, lower for ZnO synthesized using plant extract, and even lower for ZnO produced by chemical synthesis. Moghaddam et al. [68] also presented similar results, where the antioxidant efficacy of green synthesized ZnO was related to the phytochemicals bound to the surface of nanoparticles. ZnO with a large amount of phytochemicals bound to their surfaces showed the highest free radical scavenging activities. The results are in accordance with the UPF values (Table 4), as the cotton sample with the lowest antioxidant activity also exhibits the highest UV protection, meaning that the highest amount of ZnO was synthesized. The sample that was sonicated in between the in situ synthesis process (Method 3) also exhibits a high K/S value (Table 6), which additionally confirms that a lot of pomegranate peel extract was adsorbed on the surface of the fibers, resulting in high antioxidant activity.

Cotton samples where in situ synthesis of ZnO was performed (samples Method 2 to Method 4) were further analyzed for their ZnO content (Table 7). The cotton sample that exhibited the lowest UPF values (Table 4, sample Method 2) contained the lowest amount of ZnO (5.6%), which can be attributed to the aforementioned problem of the method used, since the active compounds for synthesis are partially removed from the substrate during the padding process and are not able to form higher amounts of ZnO-NP on cotton. From this point of view, the padding method is not the most suitable for an in situ synthesis of ZnO on the cotton. The cotton sample that had the highest UPF values (Table 4, sample Method 4) and the lowest antioxidant activity (Table 6, sample Method 4) really does contain the highest amount of ZnO (9.6%). This additionally confirms that Method 4, in which continuous drying of the sample between immersion in the active solutions for the synthesis was incorporated into the synthesis process, is the most appropriate method for the in situ synthesis of ZnO on cellulosic textile substrate.

**Figure 11:**
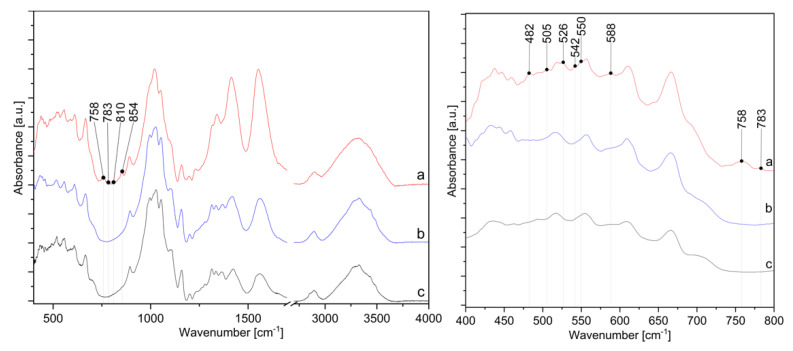
IR spectra in the range from 400 to 4000 cm^−1^ (**left**) and from 400 to 800 cm^−1^ (**right**) of cotton samples: (a) in situ synthesis of ZnO-NP with Method 4, (b) treated with zinc acetate dihydrate solution, and (c) treated with pomegranate peel extract and zinc acetate dihydrate.

The presence of ZnO-NP on the cotton sample prepared by Method 4 was further confirmed by FTIR analysis. Figure 11 shows the FTIR spectra of the cotton samples where in situ synthesis of ZnO-NP was performed with Method 4, using the sample treated with only zinc acetate dihydrate solution and sample treated with pomegranate peel extract and zinc acetate dihydrate. All spectra show characteristic bands for cellulose, with some differences in the range of 800 to 400 cm^−1^. A cellulose “fingerprint” can be observed due to the strong broad peaks at approximately 3330 cm^−1^ due to the stretching vibrations of hydroxyl (OH) groups around 2890 cm^−1^ as C–H stretching and the large band around 1020 cm^−1^ relate to C−O stretching [35,69,70,71,72]. The significant changes in the FTIR spectra are noticeable for the sample in Method 4 in the range from 1350 to 1600 cm^−1^ and from 400 to 800 cm^−1^. The intensity of the peaks at 1413 and 1552 cm^−1^ increases, which is attributed to antisymmetric and symmetric stretching vibrations of the carboxylate groups from the zinc acetate dihydrate precursor [73,74]. Since the peaks are higher for the sample in Method 4 than for Zn-acetate alone or the sample without wood ash, this confirms that the addition of wood ash extract is crucial for the high adsorption of active solutions on cotton. The sample Method 4 also contains new peaks in the FTIR spectrum at 482, 505, 526, 542, 550, 758, 783, 810, and 854 cm^−1^, which other samples, i.e., the samples treated with pomegranate peel and zinc acetate dihydrate or zinc acetate dihydrate alone, do not have. According to the literature, the appearance of absorption bands in the range of 400–500 cm^−1^ [75,76,77] or even at higher frequencies, i.e., 512 cm^−1^ [78], 568 cm^−1^ [70], and 608 to 731 cm^−1^ [79] are characteristic peaks for ZnO vibrations. As reported by Jayarambabu et al. [79], the peak at 854 cm^−1^ confirms the formation of tetrahedral coordination of ZnO. ZnO crystalizes into three main forms—hexagonal wurtzite, zinc blend, and rocksalt structure, among which the wurtzite structure is thermodynamically stable under ambient conditions [80]. Since the wurtzite structure consists of tetrahedrally coordinated O^2−^ and Zn^2+^ ions [81], the newly formed band could confirm the formation of wurtzite structured ZnO-NP.

**Figure 12:**
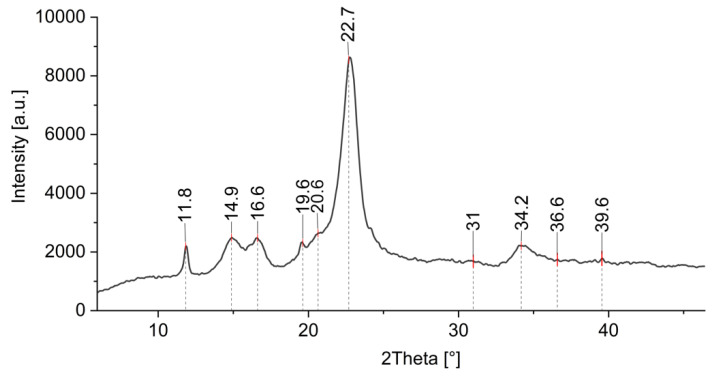
XRD pattern of cotton sample where in situ synthesis of ZnO-NP was performed with Method 4.

Additional information of formed ZnO-NP on cotton was obtained by XRD analysis. Figure 12 shows the XRD pattern of a cotton sample treated by Method 4, where ZnO-NP have been synthesized directly on the fabric using the green approach. There is an intensive peak observed at 2θ ≈ 23° and less intensive peaks at around 2θ ≈ 14°, 2θ ≈ 16°, and 2θ ≈ 22°, which are characteristic peaks for cotton [82,83,84,85]. There are several studies where cellulose decorated with ZnO was analyzed using XRD, and it was found that the peaks from 30 to 36° and even 39° are related to the ZnO crystallite [26,82,83,86,87] and can be ascribed to the (100), (002), and (101) crystal planes of ZnO [85,88,89,90]. Similar results were obtained with our samples, with the peaks around 31°, 34°, 36°, and 39°. The weak ZnO band signals were found to be in cases of lower concentrations of ZnO and higher precursor to natural extract ratios, and they also depend on the type of the natural extract and the crystalline structure of the final ZnO product [85,91,92]. The precursor type and the treatment process also lead to different nano-structured and nano-porous forms of ZnO with different coordinated networks, which exhibit different peak intensities (some very weak) in the XRD spectrum [93,94,95,96,97]. The presence of (100), (002), and (101) crystal planes in our XRD pattern confirm the wurtzite structure of the synthesized ZnO [85,98] as previously also observed by FTIR analysis. The peak at 2θ ≈ 19.6 is attributed to the presence of a minor crystalline phase of Zn–extract complexes [99].

**Figure 13:**
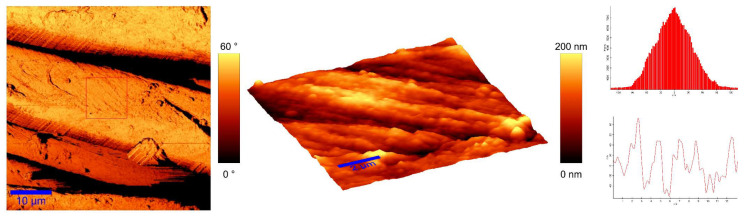
AFM images of cotton fabric functionalized with pomegranate peel extract and zinc acetate dihydrate.

**Figure 14:**
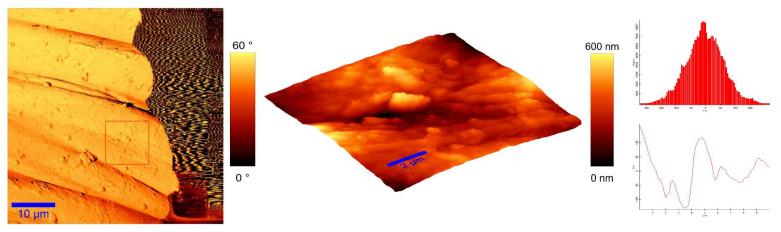
AFM images of cotton fabric functionalized with Method 4 of in situ ZnO synthesis on cotton fabric.

To identify the topological appearance, atomic force microscopy (AFM) was performed. The images of the cotton sample functionalized with pomegranate peel extract and zinc acetate dihydrate and the sample functionalized with Method 4 of in situ synthesis of ZnO are presented in Figure 13 and Figure 14. It can be observed that treatment in pomegranate peel extract and zinc acetate dihydrate results in lower roughness of the functionalized cotton, while with Method 4 of in situ synthesis of ZnO, higher roughness is achieved. The root mean square value (RMS) of the samples was calculated, and the results showed that the value increases for the sample where the in situ green ZnO synthesis process was performed. The RMS value increased from 37.6 nm (pomegranate peel and zinc acetate dihydrate treated cotton) to 96.7 nm (Method 4) due to the formed ZnO-NP on the fabric’s surface. The cotton fibers with ZnO on their surface are rougher and the coating layer thickness is higher.

## 4. Conclusions

In this study, ZnO-NP were in situ synthesized on cotton fabric using a green and circular economy approach, where Zn-acetate was precipitated with water extracts from biowaste, such as pomegranate peel and wood ash. The results showed that in the synthesis process, the use of only water extract of pomegranate peels and zinc precursor does not produce ZnO-NP on cotton’s surface, and that it is necessary to add an alkali source (in our case wood ash) for a complete formation of ZnO-NP, which was confirmed by antioxidant analysis, SEM, FTIR, XRD, and AFM. Among the four studied methods for in situ ZnO-NP synthesis, the method that included continuous drying between the immersion in the active solutions for the synthesis (Method 4) was found to be the most appropriate, as it delivered cotton fabric with excellent UV protective properties due to the uniformly distributed numerous ZnO wurtzite nanoparticles on its surface. Other methods are not recommended to be applicable due to the very stiff hand feel of the fabric and surface cracking (Method 1) or redundant (not necessary) step in the procedure, while delivering fabrics with the lowest UV protective properties, i.e., padding in Method 2 and sonication in Method 3. In situ synthesis of ZnO-NP on cotton using Method 4 has great potential to advance the development of environmentally friendly functional textiles, as it simultaneously addresses multiple problems—the use of discarded by-products of food production and pellet heating, while not requiring expensive specialized equipment (i.e., padder or sonicator), which overall reduces the environmental impact of classical chemical textile functionalization and enables the production of protective textiles that could be used in a clothing, medical, or technical textile sector.

## Figures and Tables

**Table 1:** Summary of the green ex situ and in situ syntheses of ZnO on textile substrates.

Synthesis Type (Ex Situ or In Situ)	Zinc Precursor	Reducing Agent and Its Preparation	Alkali Source	Synthesis Procedure (and Application to Textile)	Ref.
ex situ	1 M Zn-acetate	Aqueous *Acalypha indica* leaf extract (20 g/L, 2 h at 70 °C, filtration)	none	Synthesis: at 60 °C for 1 h, heating at 80 °C for 24 h, calcination at 100, 300, and 600 °C (time N/A); Application: pad-dry-cure method	[9]
in situ on cotton	0.1 M Zn-nitrate	Tragacanth gum (1%)	0.2 M NaOH	2× ultrasonication for 1 h, drying at 60 °C	[21]
partially in situ on cotton	Zn-acetate	Aqueous date seed extract (2 g/250 mL for 6h, filtration)	NaOH	Synthesized Zn-hydroxide was applied on fabric by 2× pad-dry-cure method	[22]
in situ on cotton	0.02 M Zn-acetate	Bacterium *Serratia ureilytica*	Biologically activated ammonia	Synthesis on fabric: 30, 60 and 90 min at 50 °C	[26]
ex situ	0.1 M Zn-nitrate	Aqueous *Pongamia pinnata* leaves extract (2 h at 100 °C, filtration)	none	Synthesis: 24 h of precipitation, centrifugation 15 min, drying at 80 °C, calcination at 350 °C for 3 h; Application: pad-dry-cure in 3% ZnO with crosslinker.	[27]
ex situ	0.1 M Zn-nitrate	Ethanolic *Nephelium lappaceum* peel extract	none	Synthesis: 2 h at 80 °C precipitation, centrifugation 10 min, drying at 40 °C for 8 h, calcination at 450 °C Application: pad-dry-cure method in 2% ZnO and crosslinker	[28]
ex situ	0.1 M Zn-nitrate	Aqueous *Prunus x yedoensis* leaf extract (50 g/200 mL, 30 min at boiling, filtration)	none	Synthesis: 8 h at 80 °C, washing with EtOH, calcination at 450 °C for 1 h. Application: pad-dry-cure method.	[36]
ex situ	100 mM Zn-sulfate	Actinobacteria *Rhodococcus pyridinovorans*	K_2_HPO_4_	Synthesis: 72 h at 30 °C, centrifugation, filtration, drying at 70 °C for 4 h Application: stirring fabric in ZnO solution for 30 min, drying 10 min at room temperature and 5 min at 135 °C	[39]
ex situ	0.5 M Zn-nitrate	Aqueous avocado fruit extract (10 g/150 mL, at 60 °C for 10 min)	NaOH	Synthesis: 1 h, washing with EtOH, calcination for 2 h at 400 °C Application: N/A	[40]
in situ on cotton	Zn-acetate	Aqueous *Keliab* extract (10 g/100 mL, overnight)	none	Cotton impregnation with precursor for 30 min, adding reducing agent at 90 °C for 60 min, drying at 80 °C for 30 min, curing at 150 °C for 3 min	[41]

**Table 2:** Color values (CIE L*a*b*) of the untreated cotton sample, sample treated with zinc acetate dihydrate solution, pomegranate peel extract, and samples where in situ synthesis of ZnO-NP was performed with Methods 1−4.

Sample		CIE L*	CIE a*	CIE b*
Untreated cotton		91.66	−0.27	1.01
C_4_H_6_O_4_Zn·2H_2_O treated cotton		91.95	−0.49	1.80
Wood ash extract treated cotton		88.72	0.14	5.85
Pomegranate peel extract treated cotton		70.36	7.93	34.92
Pomegranate peel and zinc acetate dihydrate treated cotton		69.38	−2.50	48.99
In situ synthesis of ZnO-NP on cotton	Method 1	60.64	4.39	51.33
	Method 2	71.36	−1.77	54.39
	Method 3	63.48	2.53	55.61
	Method 4	56.16	5.73	47.17

**Table 3:** K/S values of the untreated cotton sample, sample treated with zinc acetate dihydrate solution, pomegranate peel extract, and samples where in situ synthesis of ZnO-NP was performed with Method 1−4.

Sample		K/S Value	λ (nm)
Untreated cotton		0.04	400
C_4_H_6_O_4_Zn·2H_2_O treated cotton		0.05	400
Wood ash extract treated cotton		0.14	400
Pomegranate peel extract treated cotton		10.85	400
Pomegranate peel and zinc acetate dihydrate treated cotton		9.09	400
In situ synthesis of ZnO-NP on cotton	Method 1	12.37	400
	Method 2	9.70	400
	Method 3	11.96	400
	Method 4	10.98	400

**Table 4:** Ultraviolet protection factor (UPF), transmission of UVA and UVB radiation (T(UVA) and T(UVB)), UVA and UVB blocking and protection category of the untreated cotton sample, pomegranate peel extract treated sample, and samples where in situ synthesis of ZnO-NP was performed with Methods 1−4.

Sample		UPF	T (UVA) (%)	T (UVB) (%)	T (UVA + UVB) (%)	UVA Blocking (%)	UVB Blocking (%)	Protection Category
Untreated cotton		3.9	28.9	24.8	27.3	71.1	75.2	Insufficient
Wood ash extract treated cotton		6.9	20.7	16.5	19.2	79.3	83.5	Insufficient
Pomegranate peel extract treated cotton		88.6	1.5	1.2	1.4	98.5	98.8	Excellent
Pomegranate peel and zinc acetate dihydrate treated cotton		50.7	2.5	2.1	2.4	97.5	97.9	Excellent
In situ synthesis of ZnO-NP on cotton	Method 1	1083.9	0.2	0.1	0.2	99.8	99.9	Excellent
	Method 2	92.3	1.5	1.1	1.3	98.5	98.9	Excellent
	Method 3	118.0	1.2	0.9	1.1	98.8	99.1	Excellent
	Method 4	154.0	1.0	0.8	0.9	99.0	99.2	Excellent

**Table 5:** Total flavonoid content and total phenolic content determination (mg/100 g) of pomegranate peel extract.

Extract	TFC (CE Catechin Equivalent mg per 100 g)	TPC (GAE Gallic Acid Equivalent mg per 100 g)
Pomegranate peel extract	765.8	2911.8

**Table 6:** Average antioxidant activity (%) of untreated cotton sample, zinc acetate dihydrate-treated cotton, pomegranate peel extract, pomegranate peel extract-treated cotton, and samples where in situ synthesis of ZnO-NP was performed using Methods 2−4.

Sample		Average Antioxidant Activity (%)
Untreated cotton		0.7 ± 0.19
C_4_H_6_O_4_Zn·2H_2_O-treated cotton		0.4 ± 0.14
Pomegranate peel extract (no textile)		94.8 ± 0.03
Cotton treated with pomegranate peel extract		79.7 ± 0.01
In situ synthesis of ZnO-NP on cotton	Method 2	27.3 ± 0.06
	Method 3	40.8 ± 0.04
	Method 4	25.9 ± 0.05

**Table 7:** Zinc concentration (c_Zn_) of cotton samples where in situ synthesis was performed using Methods 2−4.

Sample		c_Zn_ (%)
In situ synthesis of ZnO-NP on cotton	Method 2	5.6 ± 0.16
	Method 3	7.8 ± 0.62
	Method 4	9.6 ± 0.33

## Data Availability

Data sharing not applicable.
